# The Sympathetic Nervous System

**Published:** 1876-04

**Authors:** S. W. Wetmore


### ART. II.—*The Sympathetic Nervous System.* By S. W. Wetmore, M. D.

[Read before the Buffalo Medical Association, Feb. 9, 1876.]

Mr. President and Gentlemen :

Hoping you will not become too "nervous" while listening to to the theme chosen for consideration this evening, I will crave your indulgence while delineating in my feeble way, how much, and yet how little we know of the

### GREAT SYMPATHETIC NERVOUS SYSTEM.

I say *great*, for such it has been called, and correctly so too ; not, however, on account of its magnitude of structure, or organization, but it is truly great in its functions, its physiological actions, and pathological conditions.

Doubtless you are as familiar and as well informed concerning its structure, arrangement, distribution, &c., as the author of this hastily prepared paper. But perhaps a brief resume may not be uninteresting, and serve to refresh our memories, and thereby enable us the better to comprehend its physiological characteristics and some of its peculiar morbid phenomena.

Suffice to say that this system of nerves consists of a series of ganglia called centres, which are connected by intermediate nerves forming a continuous cord (called the *trunk* of the sympathetic) on each side of the spine from the first cervical vertebræ to the ganglion impar, sitúated in front of the cocyx. They can also be traced up into the head, where they all commnnicate with the fifth cranial nerve. For convenience of description the ganglia are divisible into groups, according to the position they occupy.*

Thus there are 4 cephalic, 3 cervical, 12 dorsal, 4 lumbar, 6 sacral and 1 coccygeal.

The four cephalic are the ophthalmic, spheno-palatine, otic and sub-maxillary.

The opthalmic (lenticular or ciliary), is situated deeply in the orbit between the optic nerve and external rectus muscle. It throws off some ten or fifteen ciliary nerves which perforate the globe and are distributed to the tunics, and particularly to the iris

* A large number of diagrams were here referred to, illustrating the position of the ganglia in the head, neck and trunk, their minute structure, cells, fibres, and how their nerves communicate with each other, and with those of the cerebro-spinal system.

and ciliary muscle. It communicates with branches from the motor oculi and opthalmic division of the trigeminus. It also sends a very minute filament through the center of the optic nerve to the retina in company with the arteria centralis retinæ.

The second or spheno-palatine, called Meckel's, is the largest of the four and is situated in the spheno-maxillary fossa, near the spheno-palatine foramen.

It receives motor filaments from the facial, and sensitive fibres from the two spheno-palatine branches of the 2d division of the fifth.

It is distributed to the periosteum of the orbit, to the gums, to the roof of the mouth, the palate and uvula, the tonsils, the mucous membrane of the nose, the pharyngeal mucous membrane, the middle auditory meatus, the levator palati and azygos uvule muscles. The 3d, otic or Arnolds' ganglion, is situated on the inner side of the 3d division of the 5th pair, immediately below its exit through the foramen ovale. It is furnished with motor power by the facial, and filaments of sensation by the trigeminus and glossopharyngeal.

It is distributed to the mucous membrane of the eustachian tube, to the tympanic cavity, to the tensor tympani, and tensor palati muscles. Arnold claims also that by a filament from the lesser petrosal it communicates with the auditory nerve, hence it is called "otic or auricular."

The 4th, sub-maxillary, also discovered by Meckel, is situated at the lower border of the gustatory nerve on the sub-maxillary gland.

Its motor root is from the chorda tympani, a branch of the facial nerve, its sensory filaments are from the gustatory. Its branches of distribution are to the sub-lingual glands their ducts, and the mucous membrane of the mouth.

The three cervical ganglia are situated opposite the 3d, 5th and 7th cervical vertebræ respectively, and are called the superior, middle and inferior ganglia. When the middle is wanting, the inferior is found fused with the 1st thoracic ganglia.

These ganglia are connected by the trunk of the so-called "sympathetic cord." They also communicate freely with the cranial ganglia, with the 3d, 4th, 5th and 6th cranial nerves, and with filaments of the cerebro-spinal system. A network of interlacing fibers cover the branches of the external carotid and vertebral arteries. They also accompany the pneumo-gastric, hypoglosal, and glossopharyngeal nerves, forming the pharyngeal plexus, sending filaments to the thyroid gland, the pharynx, larynx, organs of voice, trachea, œsophagus, heart and lungs.

Beneath the arch of the aorta the plexus is joined by the nerves given off from the inferior cervical ganglion, viz: the inferior cardiacs. These nerves communicate freely with the recurrent laryngeal.

### THORACIC GANGLIA.

There are usually twelve, though sometimes two are fused into one.

They are situated behind the pleura, over the head of each rib.

The first is the largest. Each ganglia receives branches from the corresponding spinal nerve.

The nerves proceeding from the ganglia supply the thoracic, and part of the abdominal and pelvic viscera, and send a small filament to each vertebra according to Cruveilhier.

From the 1st and 2d ganglia branches are given off which join the cardiac plexus, and form the 3d and 4th, like filaments to the pulmonary plexus. While the *great splanchnic* nerve is formed by branches from the 6th to the 10th ganglion. The branches descend obliquely along the sides of the bodies of the vertebræ, and unite into a single nerve, which is a large, white, rounded cord.

It penetrates the diaphragm and passes into the *semilunar ganglion*, sending a few filaments to the renal plexus and supra-renal capsules.

The *lesser splanchnic* nerve arises from the 10th and 11th ganglia passes into the abdomen and is lost in the renal plexus, which is formed in the filamentous termination of the *smallest* or *renal splanchnic* nerve, from the last or 12th ganglion.

### THE SEMILUNAR GANGLIA.

The largest of the sympathetic, sends off from each side, radiating branches, and form the *solar* plexus. This surrounds the cæliac axis like a ring, and is called the brain of the abdomen, for it contaius a large quantity of ganglionic matter.

From this as from a root, other secondary plexuses branch off and twine around the arteries, thus forming the phrenic plexus the coronary, gastric, hepatic, splenic, superior mesentric renal, spermatic, &c.

The four lumbar ganglia are situated upon the bodies of the vertebræ. Their branches unite to form the aortic and hypogastric plexuses, and follow the course of the blood-vessels.

The five sacral ganglia and ganglion impar, send filaments to all the pelvic viscera and the blood-vessels. The most interesting ones are the *uterine* nerves, which supply that organ, and the fallopian tubes. Within the substance of the uterus collections of ganglionic cells are found to connect the nerves. These were described by Dr. Robt. Lee in 1857. Recent researches have proven the existence of ganlionic cells in connection with the terminimal filaments of this system of nerves almost everywhere particularly in the mucous membrane as well as the muscular layer of the alimentary canal, pancreas, spleen, the liver and its ducts, the salivary glands, the blood-vessels, lungs, larynx and trachea, lymphatics, bladder, ureters, the generative organs, ciliary muscle, iris and lachrymal canals.

It will be observed that they seem to have an affinity for or are distributed to organs and structures containing large quantities of *yellow elastic fibrous tissue.*

It will be remembered that this tissue enters very generally into the structure of organs in which the property of elasticity is an important quality, and, in certain situations, it is the sole tissue present.

As in the ligamenta sub-flava, chordæ vocales, thyro-epiglotic ligament, crico-thyroidean membrane the membraneous layers connecting the cartilagenous rings of the trachea and bronchial tubes, the ligamentum suspensorium penis, and the middle coat of arteries.

It is also met with in the alimentary canal, the œsophagus, and anus, and around the male and female urethra.

Can we not then account for many morbid conditions, with which we are daily brought in contact, supposed to be "reflex action," before we take up the functions of this important piece of mechanism.

Concerning the *intimate structure* of the sympathetic ganglia and nerves little need be said, inasmuch as they possess very little physiological importance. In form the ganglia are rounded, ovoid or pear-shaped. The interior is made up of nerve-cells, nerve-fibre, and connective tissue. The cells are not unlike in appearance those of the encephalon and spinal cord. Some are unipolar, some are bipolar, and some are multipolar.

The true efferent fibre which make up the sympathetic nerve is medullated (Remak), and measure from 1-10,000 to 1-5,500 of an inch in diameter.

The connection between the cells and fibres is supposed to be the same as in the cerebro-spinal centre.

As the sympathetic nerves leave the ganglia, there may be seen accompanying them both motor and sensative filaments from the spinal cord. These can also be traced to their ultimate ramifications.

They are softer and more grayish in color than the spinal nerves and hence we are able to designate the one from the other, when they communicate, by the white appearance of the latter.

### FUNCTION.

The older writers had no very clear ideas concerning the functions of this system of nerves.

It was not until 1725 that the influence of the sympathetic nerve of the neck was demonstrated to be propagated from below toward the head, and not from the brain downward, and then only so far as the eye was influenced by its division. This discovery was made by Pourfour du Petit.

In 1816 Dupuy removed the superior cervical ganglia in horses, with the effect of producing injection of the conjuntiva, increase of temperature in the ear, and an abundant secretion of sweat upon one side of the head and neck.*

* Flint.

These experiments showed the sympathetic has an important influence on nutrition, calorification and secretion.

In 1851 Bernard repeated the experiments of Petit, dividing the sympathetic in the neck on one side in rabbits, and noted on the corresponding side of the head and the ear increased vascularity and an elevation of temperatnre amounting to from 7° to 11° Fahr.

In 1852 Brown Séquard repeated these experiments, with like results, and attributed the elevation of temperature directly to an increase in the supply of blood to the parts affected. He also demonstrated that its section paralyzed the muscular walls of the arteries, and further, that galvanization of the nerve of the neck caused the vessels to contract.

This was the discovery of the vaso-motor nerves."

Soon after, however, Bernard performed analogous experiments, and after having divided the sympathetic in the neck of a large rabbit, cut off with a sharp knife both ears just above the head, and found that the artery on the side on which the nerve was divided, would send up a jet of blood several feet high, while on the sound side it was much less forcible, and not unfrequently was not observed at all.

The influence of the sympathetic over the secernents are very marked, as shown by Peyrani, Bernard, Séquard, Moreau and others, who have paralyzed the sub-maxillary filaments increasing the salivary secretions.

It appears that galvanizing the sympathetic increases the amount of urine and urea, and is greater with the induced than the constant current.

"Dr. Moreau published in 1860 a very elaborate and important series of experimental observations on the influence of the sympathetic nerves upon the secretion of liquid by the intestinal canal, which are peculiarly interesting in their bearing upon the sudden occurence of watery diarrhea."*

It can only be through the sympathetic nerves that the states or conditions of the mind can influence the muscular apparatus of organic life.

There are many organs that are influenced on the one hand by

* Flint.

emotional states, and on the other by the state of expectant attention. The heart sympathizes probably more with the emotions than any other organ in the body. The physician can very often infer this while feeling the pulse, its beats frequently being audible.

The alterations in the diameter of the blood-vessels is wholly under the vaso-motor system or "nervi-molles," as is evinced in blushing, by the emotion of modesty or shame, or to the pallor which alternates with this, in many states of mental agitation."

"The influence of particular conditions of the mind in exciting suspending or modifying various secretions is a matter of daily experience—the *lachrymal* secretion, for example. The flow of *saliva* is stimulated by the sight, the smell, the taste, or even the *thought* of food, especially of such as is of a savory character."*

The same may be said of the tendency to micturition, not unfrequently fear, or anxiety, act as a powerful diuretic.

The peristaltic action of the intestines are often increased by fear or expectant attention.

Dr. Carpenter relates a case of a "Lecturer in a public institution who was always seized with a strong impulse to defecation during his lecture, and was greatly inconvenienced by the effort to restrain it."

The uterus and the generative organs are greatly influenced by certain states of the mind. We are all as accoucheurs familiar with the cessation of labor pains, on first entering the lying-in chamber, especially if we be a stranger to our patient.

There is probably no secretion that so strongly manifests the influence of the emotions, both upon its quantity and quality, as that of the mammary glands. The modus operandi is analagous to that which takes place in the act of blushing.

Sir A. Cooper says: "A *fretful temper* lessens the quantity of milk, makes it thin and serous, and produces much griping in the child.

"*Fits of anger* produce a very irritating milk, followed by griping in the infant, with green stools.

"*Grief and anxiety of mind* have a great influence on lactation, and diminish the quantity and alters the quality of the milk.

* Carpenter.

"*Fear and terror* often instantly stops the secretion."

Carpenter says "he has positive evidence that the mammary secretion may acquire an actually *poisonous* character under the influence of violent mental excitement.," and cites cases where children have died in convulsions immediately after nursing the mother, who had met with some distressing occurrence.

From what has been said, it appears obvious that this system of nerves serves as a medium of reflex action between the motor and sensative portions of the alimentary canal, or the digestive, excretory and generative apparatusses, and it is positive that it takes part in reflex action with the cerebro-spinal nerves.

There are accordingly three different kinds of reflex actions taking place wholly or partially through the sympathetic system which may be observed to occur in the living body.

1*st.* *Reflex actions taking place from the internal organs through the sympathetic and cerebro-spinal systems to the voluntary muscles and sensitive nerves.*

The convulsions of young children are often owing to the irritation of undigestible food in the intestinal canal.

Attacks of indigestion are also known to produce temporary amaurosis, double vision, strabismus, and even hemiplegia.

Nausea and a diminished or capricious appetite are often prominent symptoms of early pregnancy, induced by the peculiar condition of the uterine mucous membrane.

2d. *Reflex actions taking place from the sensitive surfaces through the cerebro-spinal and sympathetic systems to the involuntary muscles and secreting organs.*

Imprudent exposure of the integument to the cold and wet will often bring on diarrhea.

Mental and moral impressions conveyed through the special senses will affect the motions of the heart, and disturb the processes of digestion and secretion.

Terror, or an absorbing interest of any kind, will produce a dilatation of the pupil, and communicate in this way a peculiar wild and unusual expression of the eye.

Disagreeable sights or odors, or even unpleasant occurrences, are capable of hastening or arresting the menstrual discharge, or of producing premature delivery.

3d. *Reflex actions taking place through the sympathetic system from one part of the internal organs to another.**

The contact of food with the mucous membrane of the small intestines excites a peristaltic movement in the muscular coat. The mutual action of the digestive, urinary and internal generative organs upon each other takes place through the medium of the sympathetic ganglia and their nerves.

The variations of the capillary circulation everywhere, and particularly in the different abdominal viscera, corresponding with the state of activity or repose of their associated organs, are to be referred to similar nervous influence. These phenomena are not accompanied by any consciousness on the part of the individual."

The functions, then, of the sympathetic nervous system can well be defined: to govern the circulation, calorification, respiration, secretion, execretion and nutrition.

The morbid conditions produced from or influenced by this system are very variable.

For several years last past the subscriber has given this theme a special daily thought, and has arrived at the following conclusions [which are corroborated, if not verified, by a multiplicity of cases]: That many of the morbid conditions for which we are daily consulted are reflex phenomena due to pathological changes in the sympathetic ganglia, their nerves, or their terminal cells. What these changes are it is not always easy to determine; but for the most part they are either an anemia or a hyperemia, in other words, a paralysis or an over-excitation, which may be idiopathic or traumatic.

Perhaps the most common malady due to reflex action is the so-called spinal irritation, with which we are all familiar. Full well do I remember the first *well-marked* case that I ever saw. It was in the autumn of 1862.

My patient was a lady aged 29 years, unmarried, and a perfect picture of health, large, fleshy, and weighing about 170 pounds.

* Dalton.

She had been a victim of misplaced confidence, for two years having been under the care of various Homeopaths.

She was accustomed to sudden and very severe attacks of pain in the epigastric region, which were accompanied with excessive and prolonged vomiting, that for days together prohibited her taking any food that she could retain.

During one of the attacks, I was summoned. The hypodermic use of morphia, and all the ordinary remedies, only served to paliate temporarily, and after several days of unremitting effort having proven futile, I called in council Prof. Rochester and the lamented Eastman.

Although I had made diligent inquiry and careful explorations of the vertebral, cerebral and uterine regions, the first-named gentleman, having had an extensive experience in such cases, was enabled by his well-trained finger to point to the single offending spinous process.

The oleum tiglii paint was directed to be applied twice or thrice daily, which in a short time alone cured my patient, who was completely relieved for a period of two years, during which time she became a married woman and a mother. Soon after, however, the same old train of morbid phenomena returned, but was again repelled with as much facility with the same remedy.

Succeeding years having brought like cases under my observation, I began to wonder why this particular portion of the human economy should be the seat of this peculiar irritability, and then considered the tissues of which the structures that reflected over and invested the spinous processes were composed.

Knowing that the sole organization of the ligamentum nuchæ, the ligamenta subflava of the arches of the vertebræ was made up of *yellow elastic fibrous tissue*, and that each vertebræ was supplied with a sympathetic nerve from the thoracic and abdominal ganglia, I concluded that the fibre-cell, or what Kolliker describes as the stellate branching corpusules, denominated by Virchow connective tissue corpusules, of which it is composed, had something to do with its causation. Then the instances of these cells in the body—which have already been given—were considered, and very readily I found that these were just the localities; that they composed the structures and organs most frequently involved in reflex-action.

When we take into consideration that the nerves of organic or vegetative life are distributed principally to the intrinsic muscles or to non-striated muscular fibre, over which the *will* has little, if any, control, we can the better understand how reflex-action occurs when their centers or ganglia are abnormally disturbed.

Unfortunately for science, though very fortunately for the human race, the cases are rare in man of traumatic injury confined to the sympathetic.

One case, however, has been reported recently by Mitchell,—where a gunshot wound in the neck produced contraction of the pupil, and flushing of the face of the same side.

Several cases of unilateral sweating of the head have recently been reported, supposed to have been produced by aneurism.

Several cases of temporary deafness have come under my observation that could only be accounted for by some disturbance in the function of the vaso motor nerves surrounding the auditory artery, or some abnormal condition of the otic or spheno-palatine ganglia.

Temporary loss of vision is doubtless not unfrequently due to some change in the ophthalmic ganglia, or to the centralis retinæ nerve.

Those cases of sudden ecchymosis of the conjunctiva, coming on, perhaps, of a night, without any known cause, can in some instances only be accounted for by some change in the nerves from the cervical ganglia, probably from cold, or an enlarged lymphatic gland.

I have a case of loss of the sense of smell at this writing, Jan. 18th, which has existed for some months, but is improving rapidly, while being treated for a congested and ulcerated condition of the cervix uteri.

The cases of *aphonia* are numerous, dependant upon uterine irritation, and doubtless my friend Prof. White can enumerate them by the scores.

Although the organs of voice are supplied by the cervical ganglia, they communicate freely with the recurrent laryngeals, branches of the pneumo gastrics, and with the entire chain of the sympathetic ganglia.

Considering that the chordæ vocales, thyro-epiglotic crico-thyroid membrane, the membraneous layer connecting the cartilaginous rings of the treachea and bronchial tubes, and the œsophagus, are composed *principally of yellow elastic tissue*, can we not the more readily account for the "globus-hystericus," so common in hysteria, another form of reflex action?

A very peculiar form of this disease has come under my observation several times during the past year. The patient, married, aged 24 years, has never borne children, has no uterine, spinal or cerebral trouble. Menstruation normal, as to time, quantity and quality, and without pain.

The paroxysms are characterized by great distention of the stomach, so much so as to enable the observer to trace its outline and form distinctly. This condition usually continues from one to three days, great pain accompanies it, and the stomach is extremly sensitive on pressure.

The distension is not produced by flatus, but there appears to be a phlogistic condition.

The case has been under the care of the best of the fraternity in Chicago, and other cities. She claims never to have been benefitted by any treatment, until galvanism was applied over the origin of the great splanchnic plexus. This controls the paroxysms and, without doubt, prohibits frequent attacks.

Several cases have recently come to my notice where the heart's action during labor has been very much interfered with, two of which I will detail, as they were extreme cases:

Case I.—October 5th, 1875, I was called to see Mrs. D——, aged 22, first child. Labor had hardly commenced, pulse *twenty-seven* (27) per minute, skin cool. An examination of the heart revealed no abnormal organic condition. She had never had rheumatism, and had always enjoyed good health.

The labor progressed favorably, and terminated in eleven hours by the use of the forceps, without chloroform. The pulse continued nearly the same for five hours after delivery, when they began to increase gradually, and twenty-four hours after they were found to be 64, their normal condition.

CASE II.—October 28, 1875, was called to Mrs. S——, aged 24, in labor with her first child; by digital examination found the os dilated not larger than a five-penny piece; cephalic presentation, and everything normal save the pnlse, which counted *one hundred and forty-eight* (148) per minute, and continued the same throughout the labor; and four days after, when it resumed its normal condition, *seventy-two* (72) per minute.

How can we account for these extremes, apparently from the same cause, and yet different effects, other than that they were produced by some morbid conditions of the sympathetic ganglia, probably the semilunar or uterine plexus? What the pathological condition was is difficult to determine. It is probably "one of those things that no man can find out."

Within a few weeks I have treated a case of persistent diarrhea, following a mild case of enteric fever, with galvanism, with success, after all ordinary remedies had failed (including argent. nitras and the vegetable astringents).

The diarrhea continued some seven weeks after the patient left the sick-room, and after the third application, he reported himself well.

Many cases might be cited elucidating the influence of the sympathetic nerves on diseases existing on account of perverted nutrition, among which might be enumerated other forms of diarrhea, marasmus, atrophy, ulcers, gangrene, and diseases of the scalp, baldness, and the sudden changing of the color of the hair by fright, terror and great anxiety of mind.

A single well-marked case of the latter having come under my personal observation, I will briefly narrate it:

Mrs. B——, a particular friend of my family's, aged 32, residing at Niagara Falls, having missed her little boy, seven years of age, and believing he had fallen over the bank into the rapids below, became so terror-stricken that for a time she lost her consciousness. The next day, towards evening, the body of the boy was found in the whirlpool. The sad facts were conveyed tc the heart-broken mother, who was still in a semi-conscious condition. The following morning her hair was found to have changed from a *black* to a glossy white, and remains the same to this day, eleven years having elapsed since the occurrence. Her eyebrows, however, remain unchanged.

Trusting that a sufficient number of cases have been cited, and that the functions and physiological actions of this organic system of nerves have been sufficiently delineated to establish beyond cavil or doubt that these abnormal conditions may arise from morbid conditions of the ganglia or nerves, and without traumatic cause, I will close, after a word concerning the therapeutical indications.

Obscure as the modus operandi may appear, I have as much confidence in galvanism, or galvano-magnetism, in some cases, and counter-irritation in others, as I have in quinine in intermittant fever, and mercury and iodide potash in syphilis.

Doubtless, you are all familiar with the fiery ordeal through which the Hon. Charles Sumner passed, under the care of Brown Séquard, in Paris, in 1857. In which case the moxa was used a half-dozen times without chloroform.

The little heroine Clara Morris was also treated in a like manner.

Few cases require the moxa. The most of them will yield "certeris paribus" to milder forms of counter-irritation.

The cases indicating the application of galvanism are very variable, some yielding readily to the friction by the hands of the "rubbist," the charlatan, "the seventh son of the seventh son," etc., etc., others requiring a current sufficient in force to paralyze the *par vagum*, and kill your patient by stopping the heart's action, which Sequard cautions us against, saying that "he came near killing his best friend once, while galvaniziug the cervical ganglia for headache."

That many remarkable recoveries have occurred in the hands of the "friction doctor," cannot be doubted by any physician of ordinary observation.

But the key to their success lays not in their peculiar endowment of electric generation, but the influence over the vaso-motor nerves, which *anybody* can produce by a well-directed effort.

Expectant attention has no little power over the remedial influence of the "frictionist," and if he is successful in keeping his patient alive to a *confident expectation* of a cure, the malady will not unfrequently succumb.

In this hasty, hurried and imperfectly-demonstrated system of sympathetic nerves, I do not claim to have made any new discoveries; but if I have made more lucid the rationalle of its morbid phenomena, then I have attained my object, if not, it is labor lost to science.

Trusting, however, that I have stimulated your disposition to inquiry, and that your vigilance will "pari passu" with your opportunities, I respectfully submit these suggestions.

